# Hyperkalemia from Dietary Supplements

**DOI:** 10.7759/cureus.859

**Published:** 2016-11-02

**Authors:** Vivek Batra, Vipin Villgran

**Affiliations:** 1 Johns Hopkins Community Physicians, The Johns Hopkins Hospital

**Keywords:** hyperkalemia, chronic kidney disease, supplements, arrhythmias

## Abstract

Hyperkalemia is a common electrolyte problem in patients with chronic kidney disease. It is typically caused by medications in patients with poor kidney function. Patients with comorbodities such as heart failure and diabetes are predisposed to electrolyte problems. Salt substitutes and dietary supplements are uncommon causes of hyperkalemia, but we propose that they are under-recognized and underdiagnosed causes in patients with chronic kidney disease. Our case report and literature review illustrates that a careful dietary history is essential in patients presenting with electrolyte disorders, especially hyperkalemia.

## Introduction

Hyperkalemia is a serious medical condition requiring early recognition and treatment. Symptoms of hyperkalemia are non-specific, including muscle weakness, malaise, and palpitations. If untreated, it can lead to life-threatening arrhythmias and cardiac arrest. Age, diabetes, chronic kidney disease, heart failure, and medications such as nonsteroidal anti-inflammatory agents, angiotensin-converting enzyme (ACE) inhibitors, beta-blockers, and mineralocorticoid receptor antagonists are strongly associated with hyperkalemia. Normally, humans require 2000 to 3500 mg per day (50–90 mEq) of potassium if the kidney function is normal. Salt substitutes and dietary supplements pose a real danger in patients with chronic kidney disease since they can cause severe hyperkalemia.

## Case presentation

A 68-year-old male from Nepal, with recent diagnosis of nonischemic cardiomyopathy, chronic kidney disease Stage II, insulin-dependent diabetes mellitus, and hypertension was admitted to the hospital for recurrent hyperkalemia (three separate hospitalizations in one month).

During the first admission, his serum potassium was noted to be 6.7 mmol/l with serum creatinine of 1.1 mg/dl. This was attributed to his cardiac medications (angiotensin-converting enzyme inhibitor and mineralocorticoid receptor antagonist), which were stopped, and he was advised a low salt diet with restricted fluid intake. On routine laboratory workup at his primary care physician about a week later, he was found to have recurrent hyperkalemia (7.2 mmol/l, serum creatinine of 1.1 mg/dl). Since he had electrocardiogram (ECG) changes, he was treated with calcium gluconate, along with medical treatment consisting of sodium polystyrene suspension and insulin/dextrose.

On his third admission, his potassium was noted to be 6.9 mmol/l with serum creatinine of 1.1 mg/dl. A detailed medical history revealed that his medications included aspirin, beta blocker, loop diuretic, insulin, statin, and metformin. He denied any recent use of nonsteroidal anti-inflammatory agents and admitted to a low sodium and potassium diet. His cardiomyopathy was well compensated, and he did not any have dyspnea, paroxysmal nocturnal dyspnea or leg edema. After stabilizing and normalizing his potassium (similar treatment as noted above), he was admitted for further laboratory investigation.

The laboratory investigation was notable for serum creatinine of 1.1 mg/dl, with a blood urea nitrogen of 35 mg/dl, serum sodium of 133 meq/l, serum osmolality of 286 mOsm/kg, magnesium of 1.6 mg/dl, calcium of 9.4 mg/dl, hemoglobin of 10.4 g/dl, white blood cell count of 5 k/mm3, platelets of 220 k/mm3, urinary pH of six, urinary sodium of 102 meq/l, urinary potassium of 39.1 meq/l, and urinary creatinine of 76 mg/dl. Transtubular potassium gradient (TTKG) was estimated to be above seven. The serum creatinine phosphokinase, renin, aldosterone, cortisol, uric acid, and phosphorous levels were within normal limits. Hemoglobin A1c was 8.1%, with 2+ proteinuria noted. Using the Modification of Diet in Renal Disease (MDRD) equation, the glomerular filtration rate was calculated around 70 ml/min/1.73 m2. ECG showed QTc interval of 421 ms and prolonged PR interval of 244 ms on his third admission.

On further questioning the patient during the course of the hospitalization, the patient admitted to using salt substitute for the past few weeks. The salt substitute he was consuming had 610 mg of potassium in ¼ teaspoon (1.2 g). In this particular patient, who had pre-existing cardiomyopathy, chronic kidney disease Stage II, and concomitant beta blocker use, the salt substitute led him to severe hyperkalemia and multiple admissions. A careful dietary history including use of supplements, dietary substitutes, herbal medications, and over-the-counter products is essential in patients presenting with electrolyte problems. Informed consent was obtained from the patient for this study.

## Discussion

Severe hyperkalemia, defined as plasma potassium greater than 6.0 mM, can lead to life threatening arrhythmias. Impaired kidney excretion is typically the cause of hyperkalemia. Drugs (nonsteroidal anti-inflammatory agents, angiotensin-converting enzyme inhibitors, mineralocorticoid receptor antagonists, and beta blockers) can also cause hyperkalemia in the appropriate setting. Patients with diabetes, chronic kidney disease, and heart failure are particularly susceptible to hyperkalemia. Dietary supplements such as salt substitutes are rare causes of hyperkalemia, though they can be potentially life threatening. Even cardiac arrest has been noted from exogenous potassium supplements [1].

A literature review shows that salt substitutes and muscle-building supplements both can cause severe hyperkalemia [2]. Patients are typically unaware of the side effects of these products, and hence clinicians need to be vigilant to advise patients with chronic kidney disease to avoid these supplements. Potassium toxicity might cause generalized weakness, paralysis, nausea, vomiting, and ileus, but in the majority of cases it presents asymptomatically.

Hyperkalemia is often discovered on routine laboratory monitoring or classic ECG changes [3]. The ECG findings in hyperkalemia include tall T waves, prolonged PR interval, shortening of QT interval, and reduction in amplitude of P waves. Ventricular rhythms (sine waves) with wide complex QRS complexes, ventricular fibrillation and asystole might be seen in untreated or severe hyperkalemia cases [4].

There have been reported cases of hyperkalemia from the use of salt substitutes in patients who have been on ACE inhibitors. Interestingly, the serum potassium returned back to baseline after stopping the salt substitutes. The treatment suggested in these cases is to stop the salt substitute and not erroneously withdraw the ACE inhibitors in the long term, given the cardio-renal protection of ACE inhibitors [5]. Salt substitutes contain about 70 mEq/teaspoon of potassium chloride [6]. The daily intake of potassium in a patient with normally functioning kidneys is recommended around 2000 to 3500 mg per day (50 to 90 mEq) [7]. Normal kidneys can maintain potassium balance if the intake is increased to 500 mEq/d slowly over a prolonged period. This ability of the kidneys to handle a lethal potassium dose is called K+ adaptation [8]. Impaired kidneys cannot handle excess potassium acutely and hence consumption of salt substitutes can lead to hyperkalemia.

Treatment of hyperkalemia from overdose due to ingestion of potassium salt substitutes includes calcium chloride for cardiac membrane stabilization if ECG changes are noted, dextrose and insulin in water, and correction of acidosis with sodium bicarbonate solution. The modalities help in controlling acute arrhythmias. Excess potassium is removed either via ion-exchange resins or mechanically via hemodialysis [9].

Though it is rare, fatal hyperkalemia has occurred from the use of salt substitutes. In the majority of cases in the literature where hyperkalemia has occurred, it is due to massive ingestion of potassium supplement in suicidal patients with normal kidney function. Patients who have impaired renal function or heart failure are at even greater risk for life-threatening hyperkalemia. A case report highlights that one tablespoon of Nu-Salt was enough to produce fatal hyperkalemia in a suicidal patient with normal renal function [10].

## Conclusions

A careful history is essential to elucidate the cause of hyperkalemia. Though historically thought to be a rare cause of hyperkalemia, we propose that salt substitutes are an under-recognized and underdiagnosed etiology contributing to hyperkalemia in patients with chronic kidney disease. Some cardiac and blood pressure medications further compound the hyperkalemia, causing the “perfect storm”; and hence, dietary history is essential in patients presenting with electrolyte problems.
